# The Association between Trimethylamine N-Oxide and Its Predecessors Choline, L-Carnitine, and Betaine with Coronary Artery Disease and Artery Stenosis

**DOI:** 10.1155/2020/5854919

**Published:** 2020-08-13

**Authors:** Fei Guo, Jun Zhou, Zhenyu Li, Zaixin Yu, Dongsheng Ouyang

**Affiliations:** ^1^Department of Clinical Pharmacology, Xiangya Hospital, Central South University, Changsha, China; ^2^Institute of Clinical Pharmacology, Central South University, Hunan Key Laboratory of Pharmacogenetics, Changsha, China; ^3^Engineering Research Center of Applied Technology of Pharmacogenomics, Ministry of Education, Changsha, China; ^4^National Clinical Research Center for Geriatric Disorders, Changsha, China; ^5^Hunan Key Laboratory for Bioanalysis of Complex Matrix Samples, Changsha Duxact Biotech Co., Ltd., Changsha, China; ^6^Department of Cardiology, Xiangya Hospital, Central South University, Changsha, China; ^7^Department of Geriatric Medicine, Xiangya Hospital, Central South University, Changsha, China

## Abstract

**Background:**

Trimethylamine N-oxide (TMAO) and its predecessor products, choline, L-carnitine, and betaine, were reported to be associated with cardiovascular events risk. However, the association of TMAO and its predecessors with extent of artery stenosis in coronary artery disease (CAD) and in different gender is still unknown. Our aim is to investigate the association of plasma TMAO and its predecessors in CAD and extent of artery lesion in different gender.

**Methods:**

94 CAD patients and 75 healthy controls (CON) were enrolled. Fasting plasma TMAO, choline, L-carnitine, and betaine were detected using liquid chromatography-tandem mass spectrometry.

**Results:**

Elevated plasma TMAO but not choline, L-carnitine, or betaine was observed in CAD (1.46(0.8–2.32) *μ*M) and severe artery stenosis patients (S) (1.62(0.91–2.81) *μ*M) compared with controls and mild artery stenosis (M) (1.18(0.67–1.7) *μ*M in CON; 1.27(0.77–1.82) *μ*M in M, *p* < 0.05). TMAO was an independent risk factor of CAD and severe artery stenosis (CAD : OR = 1.81, 95%CI: 1.07–3.09, *p*=0.03; S : OR = 1.36, 95%CI: 1.01–1.84, *p*=0.04). TMAO was more sensitive in diagnosing CAD and severe artery stenosis from controls in men rather than in women by ROC analysis (AUC for men and women in CAD: 0.64 versus 0.57; AUC for men and women in S: 0.64 versus 0.58), while the combined four metabolites greatly improved diagnostic accuracy in women with CAD and severe artery stenosis (AUC in CAD: 0.64, AUC in S: 0.68).

**Conclusion:**

The associations of TMAO with CAD and severe artery stenosis were sex-related. TMAO alone was more powerful in determining CAD and artery stenosis in men than women, while a combination of TMAO, choline, L-carnitine, and betaine could be potential biomarkers for diagnosing CAD and artery stenosis in both men and women.

## 1. Introduction

The prevalence of coronary artery disease (CAD) is growing rapidly nowadays, which makes it the leading cause of death in many developed and developing countries. CAD is responsible for 17.7 million deaths, which account for 31% of all global deaths every year. In China, the overall number of estimated prevalent cases of CAD was 93.8 million according to recent data, which resulted in about 4 million deaths annually [1]. Traditionally, CAD is strongly associated with age, obesity, diabetes, and hyperlipidemia. Even sufficient attention was devoted to those traditional risk factors, prevalence rate for CAD still increased remarkably by 14.7% from 1990 to 2016 [2–4]. Particularly, some individuals with severe artery stenosis (≥90% in coronary arteries) do not exhibit obvious clinical symptom, which may hinder the diagnosis and prevention of CAD. Given this unfulfilled need for deeper understanding and effective preventive for CAD, uncovering new risk factors and biomarkers for CAD is of great importance.

Recently, multiple studies have suggested strong association of gut microbiota with CAD pathogenesis and progression [4, 5]. The gut microbiota is influenced by dietary intake and in turn produce metabolites that contribute to affecting the metabolism and immunity of the host. Remarkably, trimethylamine N-oxide (TMAO), a gut microbial metabolite, was indicated to be a proatherogenic factor. Study conducted by Hazen firstly reported elevated TMAO in both atherosclerosis patients and mice model in 2011 [5]. Afterwards, several studies indicated that elevated TMAO level could predict an increased risk of major adverse cardiovascular events [6–8]. TMAO arises from gut microbiota-mediated metabolism of dietary trimethylamine-containing compounds including choline, betaine, and L-carnitine. Those predecessor products of TMAO were also suggested to be associated with atherosclerosis and/or myocardial ischemia risks. Dietary supplement with choline could enhance atherosclerosis in mice [9]. Plasma level of L-carnitine, which is abundant in red meat, contributed to the prediction of increased risks of cardiovascular disease and major adverse cardiac events [10]. A study which involved 3924 African-American participants indicated higher risk of CAD incident with higher dietary betaine intake [11]. However, the relationship between plasma TMAO, choline, L-carnitine, and betaine levels and extent of arterial stenosis of cardiovascular in CAD patients has not been investigated, and whether the combination of TMAO and its predecessors could help to diagnose CAD and predict the risk of severe stenosis in different gender is still unknown. In this study, we aimed to explore the relationship between plasma TMAO, choline, L-carnitine, and betaine concentrations with CAD incidence and severity of arterial stenosis in male and female CAD patients.

## 2. Materials and Methods

### 2.1. Study Population

We recruited 73 healthy controls (CON group) and 94 CAD patients (CAD group) who were hospitalized for coronary angiography. At least one main coronary artery with luminal stenosis diameter ≥ 50% (determined by quantitative coronary angiogram analysis) was diagnosed as CAD (18 ≤ age ≤ 75 years). Age- and sex-matched CON group was an independently recruited set who underwent coronary artery CT or coronary angiography for chest pain but showed negative results. The CAD group was further divided into severe artery stenosis group (S, *n* = 45) and mild artery stenosis group (M, *n* = 49). Severe artery stenosis was defined as ≥90% stenosis in at least one of the main coronary arteries.

We exclude subjects with an active infection, malignancy, severe liver or heart cerebrovascular diseases, and severe proteinuria (>3.5 g/day) and those who received probiotics or antibiotic treatment within 1 month of enrollment to minimize potential confusing factors.

Study subjects underwent complete clinical history and physical examination in the study duration. General information including age, sex, weight, and body mass index (BMI) was retrospectively collected from each subject's medical records. All subjects were informed before being enrolled in the study.

### 2.2. Laboratory Test

Fasting blood samples (5 mL) were collected using EDTA-K treated tubes. Blood samples were centrifuged at 1000 ×*g* for 10 min and stored at −80°C until analysis. Plasma TMAO, choline, L-carnitine, and betaine were measured by high-performance liquid chromatography-tandem mass spectrometry (HPLC-MS) using D9-TMAO, D9-choline, D3-L-carnitine, and D9-betaine internal standards as described previously [12]. Hemoglobin (HGB), fasting blood glucose (Glu), creatinine (Cr), triglyceride (TG), total cholesterol (TCHO), alanine aminotransferase (ALT), glutamic-pyruvic transaminase (AST), low-density lipoprotein (LDL), and high-density lipoprotein cholesterol (HDL-C) were measured using HITACHI7170S automatic biochemical analyzer. Blood pressure (systolic blood pressure (SBP) and diastolic blood pressure (DBP)) and heart rate (HR) were measured after rest for at least 15 min.

### 2.3. Statistical Analysis

Statistical presentation and analysis of the current study were performed using the computer SPSS program (Statistical Package for the Social Science, Chicago). Categorical variables are presented as numbers and percentages. Continuous data are presented as means ± standard deviations (SD) for normal distribution parameters or medians (interquartile ranges, IQR) for nonnormal distribution parameters to investigate the dynamic change of TMAO and its predecessors in study cohort. Student's *t-*test or the Mann–Whitney *U* test was used for differences evaluation between two groups. Logistic regression analysis was performed to examine the odds ratio (OR) and 95% confidence interval (95% CI) of TMAO and its predecessor products for CAD and severe artery stenosis in male, female, and all participants; adjustments were made for variables including age, gender, BMI, SBP, DBP, Glu, TG, and Cr. Because the distribution of TMAO, choline, L-carnitine, and betaine was skewed, they were log-transformed in logistic analysis. The area under the receiver-operating characteristic curve (AUC) was calculated to evaluate the value of betaine, choline, L-carnitine, and TMAO in predicting the risk of CAD and severe artery stenosis. A two-tailed *p*-value < 0.05 was considered statistically significant.

## 3. Results

### 3.1. Patients Characteristics

Of the 94 CAD patients and 73 healthy controls, the baseline patient characteristics are displayed in Table 1 as categorized by CAD and CON group. 42.47% of CON and 42.55% of CAD group were male. A comparison of the baseline characteristics between groups with or without CAD showed that CAD patients tended to exhibit higher blood pressure (SBP = 138 ± 3; DBP = 81 ± 1 in CAD versus SBP = 128 ± 3; DBP = 75 ± 2 in CON, *p*=0.01) and to have higher Glu (5.46(4.77–6.64) mmol/L in CAD versus 4.96(4.57–5.73) mmol/L in CON, *p*=0.01) and TG (1.85(1.31–2.65) in CAD versus 1.31(0.97–1.85) mmol/L in CON, *p* < 0.01) concentrations than CON subjects. However, other factors like age, gender, height, weight, BMI, HR, TCHO, HDL, LDL, HGB, Cr, ALT, and AST were similar between two groups (*p* > 0.05, Table 1).

### 3.2. Relationship between Plasma TMAO, Choline, L-Carnitine, and Betaine Levels and CAD

**Figure 1:**
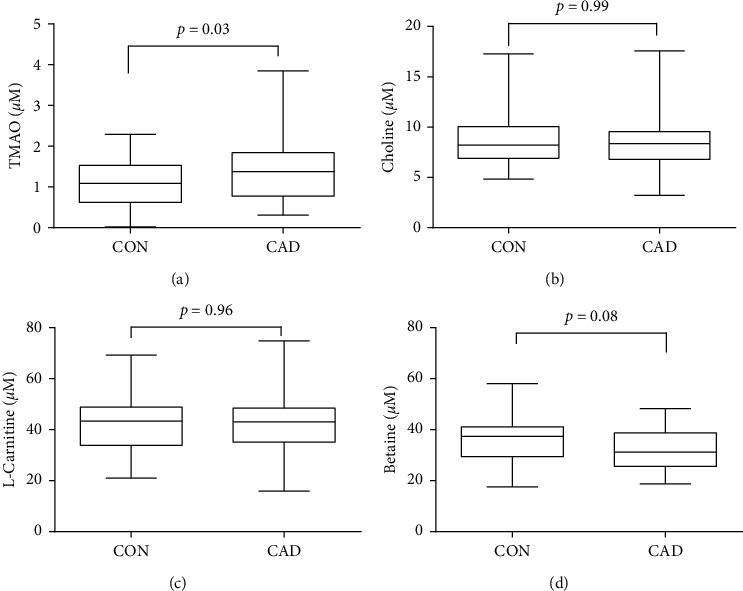
Comparison of plasma TMAO, choline, L-carnitine, and betaine levels between CAD and CON groups. Detected plasma TMAO (a), choline (b), L-carnitine (c), and betaine (d) concentration in CAD and CON group.

**Figure 2:**
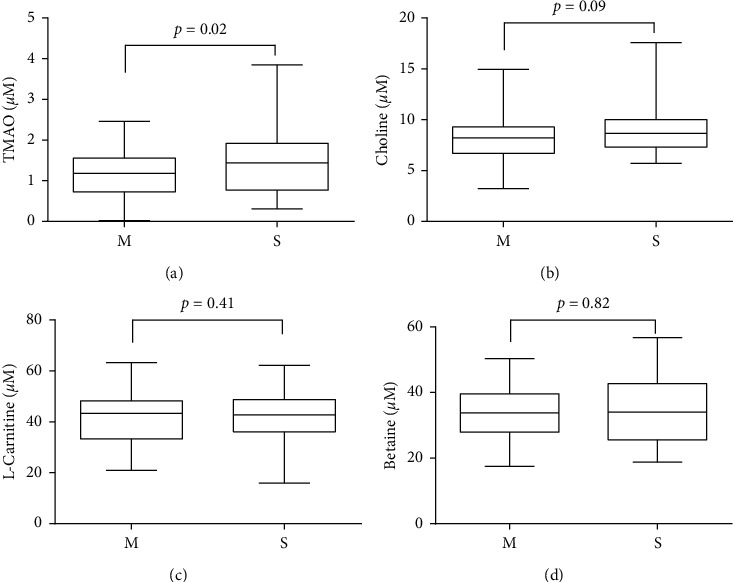
Comparison of plasma TMAO, choline, L-carnitine, and betaine levels between mild artery stenosis and severe artery stenosis. Detected plasma TMAO (a), choline (b), L-carnitine (c), and betaine (d) concentration in mild artery stenosis and severe artery stenosis groups. M: mild artery stenosis; S: severe artery stenosis.

We performed a cross-sectional comparison of TMAO, choline, L-carnitine, and betaine concentrations between CAD patients and CON group at first. We observed remarkably higher TMAO concentration in CAD group than CON group (1.46(0.8–2.32) *μ*M versus 1.18(0.67–1.7) *μ*M, *p*=0.03) (Table 1 and Figure 1). No significant difference was found in choline, L-carnitine, and betaine among CAD and CON group. Then we divided the CAD group as severe artery stenosis group (S, *n* = 45) and mild artery stenosis group (M, *n* = 49) to see if the concentrations of those metabolites could further increase in severe artery lesion patients. As showed in Figure 2, only TMAO showed significantly elevated concentration in S group compared with M group (1.62(0.91–2.81) *μ*M versus 1.27(0.77–1.82) *μ*M, *p*=0.02).

Logistic regression analysis was employed to evaluate the risk of TMAO, choline, L-carnitine, and betaine in CAD and artery lesion. Results showed that TMAO was an independent prognostic risk factor for CAD even adjusted for traditional risk factors including age, gender, BMI, Glu, TG, and Cr in model 1 (OR = 1.81, 95% CI: 1.07–3.09, *p*=0.03). We performed a second model as model 2 which includes choline, L-carnitine, and betaine and all factors in model 1. TMAO remained to be an independent risk factor (OR = 1.12, 95% CI: 1.12–3.57, *p*=0.02) while choline, L-carnitine, and betaine exhibited a very low odds ratio with no statistical significance in CAD (*p* > 0.05) (Table 2). More than that, we analyzed the risk of TMAO and its predecessors in different gender with CAD. As showed in Table 2, TMAO was independently and significantly associated with the presence of CAD after being adjusted for other traditional risk factors in male subjects (model 3, OR = 2.64, 95% CI: 1.04–6.69, *p*=0.04). When further adjusted for its predecessors, the risk of TMAO remained robust in CAD with an odds ratio of 2.58 (*p*=0.05). No significant association of TMAO with CAD incidence in female subjects was observed (model 5, OR = 1.33, 95% CI: 0.71–2.49, *p*=0.37; model 6, OR = 1.66, 95% CI: 0.76–3.65, *p*=0.2). None of choline, L-carnitine, and betaine showed statistical significance in the relationship of CAD in male or female group (*p* > 0.05) (Table 2).

Similar results were found in the association of TMAO and its predecessors with extent of artery stenosis. Significant predictive value of plasma TMAO was preserved after adjustment for traditional risk factors in total study cohort (model 7, OR = 1.36, 95% CI: 1.01–1.84, *p*=0.04; model 8, OR = 1.37, 95% CI: 1.01–1.86, *p*=0.05) (Table 3). The odds ratios of TMAO in severe artery stenosis were 1.38 versus 1.33 in male subjects and 1.25 versus 1.14 in female subjects after adjustment with traditional factors and its predecessors, but there is no statistical significance (*p* > 0.05). The association of choline, L-carnitine, and betaine with the presence of artery stenosis in total, male, and female groups was not statistically significant (*p* > 0.05).

### 3.3. Diagnostic Value for TMAO and Its Predecessors in Determining CAD and Artery Stenosis

**Figure 3:**
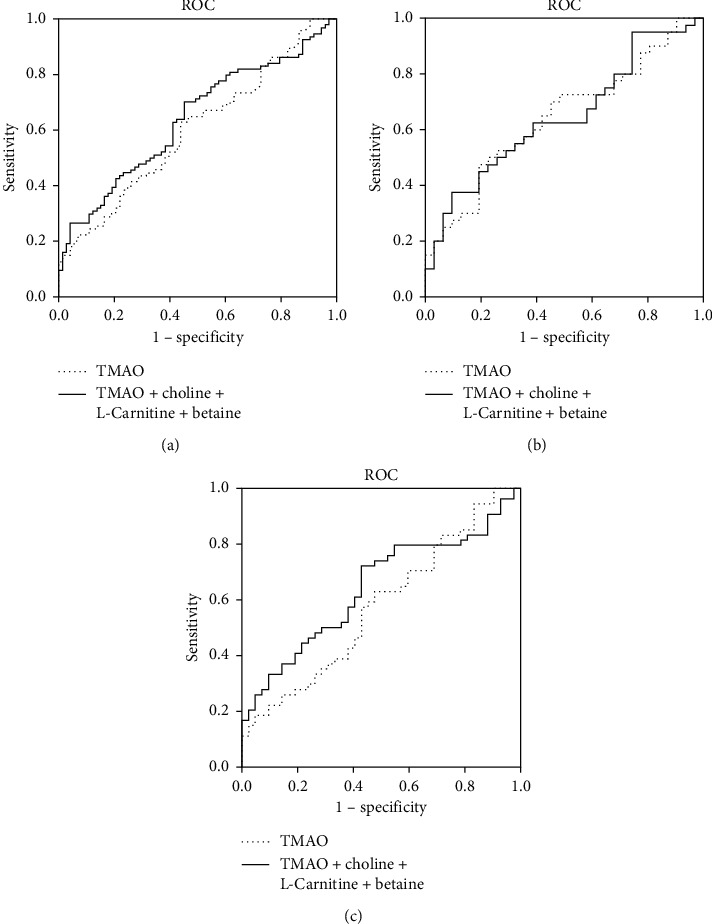
ROC curves of betaine, choline, L-carnitine, and TMAO for discriminating CAD from CON in all, male, and female participants. AUC indicates area under the receiver-operating characteristic curve. (a) All; (b) male; (c) female.

**Figure 4:**
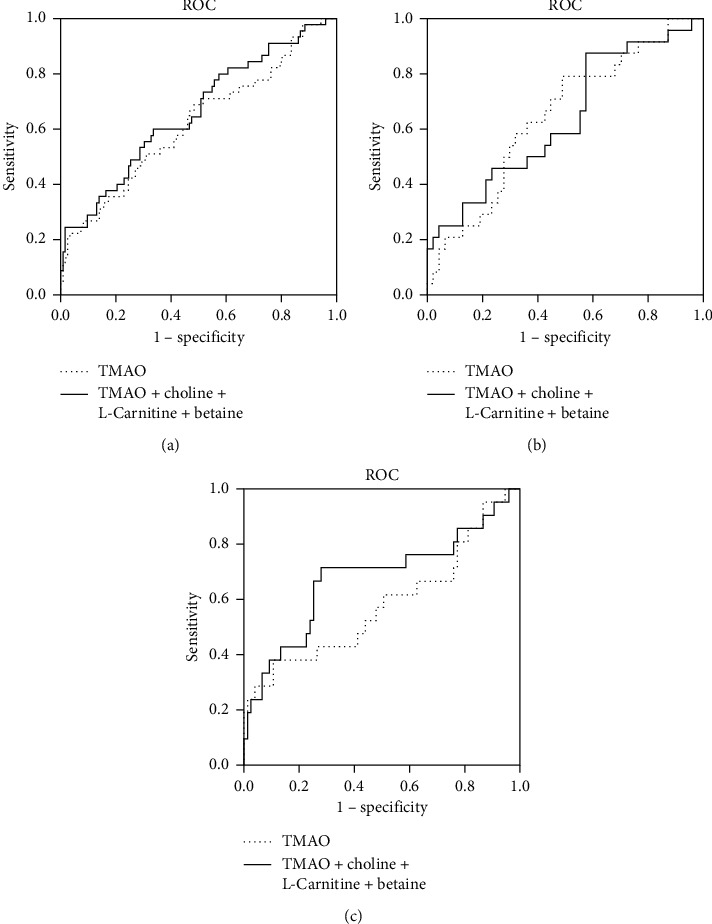
ROC curves of betaine, choline, L-carnitine, and TMAO for discriminating severe artery stenosis from mild artery stenosis in all, male, and female participants. AUC indicates area under the receiver-operating characteristic curve. (a) All; (b) male; (c) female.

To explore the role of TMAO and its predecessors in distinguishing CAD from healthy controls and severe artery stenosis from mild artery stenosis, we performed receiver-operating characteristic curve (ROC) analysis. As showed in Figures 3 and 4, the AUCs of TMAO in discriminating CAD and severe artery stenosis in total study cohort were 0.61 (*p*=0.03) and 0.62 (*p*=0.02). Combination of TMAO and predecessors showed slightly improved diagnostic accuracy with AUCs of 0.64 (*p* < 0.01) and 0.66 (*p* < 0.01) in discriminating CAD and severe artery stenosis. In male group, combination of TMAO and its predecessors or TMAO alone exhibited almost identical AUC for diagnosing CAD and artery lesion (0.64 versus 0.64, *p*=0.04 for CAD; 0.64 versus 0.64, *p*=0.05 for artery stenosis), while in female group, combination of TMAO and its predecessors exhibited better accuracy and higher sensitivity than TMAO alone at diagnosing CAD and artery stenosis extent (AUC of combined metabolites = 0.64, *p*=0.02 versus AUC of TMAO = 0.57, *p*=0.21 in CAD; AUC of combined metabolites = 0.68, *p*=0.01 versus AUC of TMAO = 0.58, *p*=0.26 in severe artery stenosis) (Tables 4 and 5).

## 4. Discussion

The main finding of the current study is (1) strong association between fasting plasma TMAO levels in patients with CAD and severe artery lesion; (2) elevated fasting plasma TMAO level which could serve as an independent predictor of CAD and the severe artery stenosis; (3) TMAO which is more accurate in diagnosing CAD and severe artery stenosis in men compared with women, while a combination of TMAO, choline, L-carnitine, and betaine showed improved accuracy in determining CAD and artery stenosis risk in both men and women.

Metabolites produced by gut microbiota could directly regulate the pathogenesis and progression of disease, among which TMAO has aroused extensive attention as it has been previously reported to directly promote proatherosclerotic effects and mechanistically involved in atherosclerosis. A case-control study indicated that TMAO was correlated with the SYNTAX score in patients with CAD [13]. Our study extends these observations by showing that plasma TMAO concentration, rather than its predecessors, was significantly associated with high atherosclerotic burden of artery (Figure 2). More than that, the logistic analysis results suggested TMAO as an independent risk factor in CAD and severe artery stenosis with odds ratios of 1.81 and 1.36 (*p* < 0.05) after adjustment for traditional factors, and the OR of TMAO slightly increased after adjustment for choline, L-carnitine, and betaine in CAD (OR = 2, 95% CI: 1.12–3.57, *p*=0.02) and in severe artery stenosis (OR = 1.37, 95% CI: 1.01–1.86, *p*=0.05) (Tables 2 and 3).

Main dietary derived predecessors of TMAO such as choline, L-carnitine, and betaine were also indicated to be involved in pathogenesis of CAD [14]. Ueland and his colleagues' study in middle and elderly men and women suggested that high plasma choline level was positively associated with cardiovascular risk factors [15]. L-Carnitine, a chemical analog of choline, was also reported to be elevated in people with CAD [16]. Koeth investigated plasma L-carnitine levels in patients undergoing cardiac evaluation and revealed risk-predicting ability of L-carnitine in both prevalent cardiovascular disease and incident major adverse cardiac events [10]. Wang's study, based on 1879 stable CAD participants, indicated that plasma betaine levels were significantly higher in CVD cases than in controls and there were dose-dependent associations with betaine concentration and the presence of CVD [5]. However, in our study, there are no obvious differences of choline, L-carnitine, or betaine concentration between CAD and CON groups as showed in Table 1 and Figure 1. Subsequently, multivariate analysis indicated that choline, L-carnitine, and betaine were not risk factors related to CAD incidence (choline: OR = 0.89, L-carnitine: OR = 0.98, and betaine: OR = 1.02, *p* > 0.05) or severity of artery lesion (choline: OR = 1.07, L-carnitine: OR = 1.01, and betaine: OR = 0.98, *p* > 0.05) (Tables 2 and 3).

Notably, the median concentrations of TMAO in CAD patients in the present study were lower than previously reported (1.46 *μ*M versus 2.2 *μ*M [17]). The median concentrations of choline and betaine in CAD patients enrolled in Ueland and Wang's study were higher than patients enrolled in our study (9.9 *μ*M versus 8.37 *μ*M for choline; 41.2 *μ*M versus 32.64 *μ*M for betaine), while the median concentration of the L-carnitine in patients reported in Koeth's study was relatively lower than patients in our study (38 *μ*M versus 43.16 *μ*M). These differences may be caused by multiple characteristics between populations such as age structure, criteria of CAD patients, composition of gut microbiota, the Eastern and Western dietary habits, and polymorphism of key metabolic enzyme. Further studies which involve all those factors are needed for more information.

There are significant differences existing between men and women in terms of disease onset, prevalence, and clinical symptoms resulting in the differences of many aspects between men and women like physiology and psychology [18]. However, most of treatment strategies were based on studies conducted on male patients. Many clinical symptoms of female patients with CAD are often atypical, leading in about 65% of women with CAD to miss the diagnosis [19]. Therefore, identifying sensitive biomarkers for different gender to reduce the incidence of CAD is of great scientific and clinical importance. The current study, to our knowledge, revealed a gender-related difference in risk-predicting accuracy of TMAO and its three predecessors in CAD and artery stenosis for the first time. Men might possess several protective physiological mechanisms with respect to TMAO production. There were studies suggested that flavin-containing monooxygenase-3 (FMO3), which was most active in TMAO synthesis pathway, was downregulated by testosterone, and the abundance of FMO3 was relatively lower in male compared to female population [20]. Despite all these, Juergen revealed a significantly lower TMAO level in female subjects than male subjects (5.4 ± 5.6 *μ*M in women versus 7.3 ± 10.0 *μ*M in men) [21]. Our study showed similar results with higher TMAO in men compared with women with CAD (1.33(0.77–2.09) *μ*M versus 1.65(1.04–2.84) *μ*M) (Supplemental Table 1). More than that, significantly elevated TMAO was observed in male CAD patients compared with male controls, but there are no significant differences of TMAO between female CAD and CON groups (Supplemental Table 1).

Results of logistic analysis in our study showed that elevated TMAO concentration tended to have higher odds ratio for both CAD incidence and sever artery stenosis in male (OR = 2.64, 95% CI: 1.04–6.69, *p*=0.04 for CAD; OR = 1.38, 95% CI: 0.93–2.04, *p*=0.11 for severe artery stenosis) than in female subjects (OR = 1.33, 95% CI: 0.71–2.49, *p*=0.37 for CAD; OR = 1.25, 95% CI: 0.74–2.12, *p*=0.4 for severe artery stenosis). None of choline, L-carnitine, or betaine showed significant risk-predicting sensitivity in CAD or severe artery stenosis in both men and women analyzed as covariant either together (Tables 2 and 3) or separately (data not shown). Interestingly, receiver-operating characteristic analysis showed that TMAO (AUC = 0.64, *p*=0.04 for CAD; AUC = 0.64, *p*=0.05 for severe artery stenosis) or combination of TMAO and its predecessors (AUC = 0.64, *p*=0.04 for CAD; AUC = 0.64, *p*=0.05 for severe artery stenosis) exhibited almost identical diagnostic accuracy and sensitivity in discriminating CAD from CON and severe artery stenosis from mild artery stenosis in male subjects. However, TMAO showed poor accuracy at discriminating CAD from CON or severe artery stenosis from mild artery stenosis in female subjects (AUC = 0.57, *p*=0.21 for CAD; AUC = 0.58, *p*=0.26 for severe artery stenosis). Nevertheless, combination of TMAO and its three predecessor products greatly improved the diagnostic accuracy and sensitivity in female subjects with an AUC of 0.64 for CAD (*p*=0.02) and 0.68 for severe artery stenosis (*p*=0.01). These differences between men and women might be related to dietary habits and microbiota composition. Several studies reported that men consume fewer fiber-rich foods and have higher red meat and fat intake compared to women [22–24], which might lead to the accumulation of trimethylamine-containing compounds and increased CVD risk in men. Evidence from microbiologists suggested that women may harbor a higher ratio of *Firmicutes*/*Bacteroidetes* in comparison to men [25, 26]. Gut bacteria in *Firmicutes* phylum are capable of producing short-chain fatty acids, which were demonstrated to contribute to CVD protection and might alleviate the risk of arteriosclerosis which arose from other gut flora metabolites like TMAO [27]. These data could provide us some hints that risk factors like TMAO alone may be less efficient in CAD risk prediction in women due to microbiota background, dietary habits, and metabolic or physiologic characteristics. When TMAO was employed for risk stratification in CAD populations, sex differences should be taken into account and assessed carefully to avoid bias in evaluations of CAD especially in women. Further investigations are needed to clarify these factors. Many cardiovascular events occur in individuals who were regarded as low-risk patients according to the currently used traditional cardiovascular risk factors due to low efficiency [28]. Jacqueline's work demonstrated improved cardiovascular risk stratification in women who were supposed at intermediate risk by adding combinations of noninvasive risk markers to traditional factors, while the risk-predicting improvements were limited in men [29]. Similarly, in our study, we did not see significant risk-predicting sensitivity of TMAO in women with respect to CAD incidence or artery lesion, but combination of TMAO, choline, L-carnitine, and betaine exhibited improved performance on CAD and artery lesion diagnosis in women. These results suggested that, regardless of the potent diagnostic role in men, TMAO itself might not be sensitive enough for CAD risk-predicting in women, but overall consideration of TMAO and its predecessors could be more appropriate for general population in CAD risk stratification.

So far, even accumulating data have indicated the link between TMAO and CVD, the causative role of TMAO in cardiovascular pathology is still under discussion. Contradictory observations also indicated that TMAO may not contribute significantly to the progression of early atherosclerotic disease risk [30]. Evidences supporting TMAO as a diagnostic marker for CAD were incoherent, and it remains unclear whether there exist factors other than diet and microbiota that may profoundly influence systemic TMAO levels. Therefore, we discussed the gender-related differences in associations of TMAO and its predecessors in CAD which might provide more information for clinical utility. However, multicenter and larger sample size will be needed to further verify these issues.

This study has several limitations. First, it is a cross-sectional study, so we cannot predict the future development of CAD. Second, the number of subjects in this study was small and selection bias cannot be excluded. And we did not include other potential confounding factors, such as patients' nutritional status, recent diet, and gut microbiota profile, which might also influence the results. Trimethylamine (TMA) is the direct precursor of TMAO, which was also suggested to be involved in cardiovascular pathology by reducing cardiomyocytes viability according to recent data [31, 32]. It would be interesting to know the associations of TMA with the CAD with regard to gender. However, the present study was designed mainly focusing on TMAO which was more stable and less volatile for our HPLC-MS-based detection method other than TMA [33]. Although we have revealed the statistically significant associations of TMAO with CAD which differed by gender, future study which involves sensitive and stable detecting methodologies for metabolites including TMA as well as FMO activity and gut microbiota background is warranted for better understanding of the potential roles of those metabolites in CAD.

## 5. Conclusions

In conclusion, the current study explored the correlation of TMAO and its three main predecessors, choline, L-carnitine, and betaine, in CAD and severe artery stenosis patients, and suggested a gender-related association of TMAO with CAD and artery stenosis. TMAO alone was powerful in risk stratification of CAD and artery stenosis in men; however, in women, no association of TMAO with risk of CAD as well as extent of artery stenosis was observed. More than that, we found that combination of four metabolites had better performance at disease diagnosis in both men and women compared with TMAO. These findings not only provided new thoughts for gender-related differences referring to onset and progression of cardiovascular disease but also suggested the potential clinical utility of the combination of TMAO, choline, L-carnitine, and betaine in artery burden stratification and CAD diagnosis.

## Figures and Tables

**Table 1:** Baseline characteristics of subjects stratified by CON and CAD.

	CON (*n* = 73)	CAD (*n* = 94)	*p*
Male (*n*, %)	31 (42.47%)	40 (42.55%)	1
Age (years)	56 ± 1	57 ± 1	0.59
Height (cm)	160 ± 1	162 ± 1	0.12
Weight (kg)	63 ± 2	66 ± 1	0.29
BMI (kg/m^2^)	25 ± 1	25 ± 0	0.5
SBP (mmHg)	128 ± 3	138 ± 3	0.01
DBP (mmHg)	75 ± 2	81 ± 1	0.01
HR	73 (66–85)	71 (66–80)	0.31
Glu (mmol/L)	4.96 (4.57–5.73)	5.46 (4.77–6.64)	0.01
TCHO (mmol/L)	4.49 ± 0.13	4.61 ± 0.12	0.71
TG (mmol/L)	1.31 (0.97–1.85)	1.85 (1.31–2.65)	<0.01
HDL (mmol/L)	1.03 (0.88–1.25)	0.96 (0.83–1.18)	0.09
LDL (mmol/L)	2.78 ± 0.09	2.83 ± 0.09	0.81
HGB (g/L)	133.5 (122–141.75)	135 (126.5–147)	0.07
Cr (*μ*mol/L)	82.89 ± 2.30	86.54 ± 2.41	0.23
ALT (U/L)	19.1 (15.1–29.48)	21.1 (15.4–32.7)	0.3
AST (U/L)	22.93 (18.35–29.1)	20.8 (18.05–31.5)	0.92
TMAO (*μ*M)	1.18 (0.67–1.7)	1.46 (0.8–2.32)	0.03
Choline (*μ*M)	8.30 (6.90–10.14)	8.37 (6.76–9.56)	0.99
L-Carnitine (*μ*M)	43.08 (33.81–49.51)	43.16 (35.44–48.94)	0.96
Betaine (*μ*M)	37.84 (29.44–42.14)	32.64 (26.11–41.33)	0.08

Continuous data are presented as mean ± SD or median (interquartile range), and categorical variables are presented as counts and percentage (%).

**Table 2:** Association of TMAO, choline, L-carnitine, and betaine levels with CAD in all, male, and female participants.

All						
	Model 1			Model 2		
	OR	95% CI	*p*	OR	95% CI	*p*

TMAO	1.81	1.07–3.09	0.03	2.00	1.12–3.57	0.02
Choline				0.89	0.7–1.13	0.33
L-Carnitine				0.98	0.94–1.03	0.47
Betaine				1.02	0.95–1.1	0.55

Male						
	Model 3			Model 4		
	OR	95% CI	*p*	OR	95% CI	*p*

TMAO	2.64	1.04–6.69	0.04	2.58	0.99–6.74	0.05
Choline				1.00	0.68–1.48	0.99
L-Carnitine				1.00	0.97–1.04	0.81
Betaine				1.00	0.91–1.1	0.98

Female						
	Model 5			Model 6		
	OR	95% CI	*p*	OR	95% CI	*p*

TMAO	1.33	0.71–2.49	0.37	1.66	0.76–3.65	0.20
Choline				0.85	0.64–1.13	0.27
L-Carnitine				1.01	0.96–1.06	0.71
Betaine				0.96	0.89–1.03	0.21

OR, odds ratio. 95% CI, 95% confidence interval. OR shown were for plasma TMAO, choline, L-carnitine, and betaine in all, male, and female participants. Models 1, 3, and 5 adjusted for age, gender, BMI, Glu, TG, and Cr. Models 2, 4, and 6 adjusted for all factors in models 1, 3, and 5 plus choline, L-carnitine, and betaine.

**Table 3:** Association of TMAO, choline, L-carnitine, and betaine levels with severe artery stenosis in all, male, and female participants.

All						
	Model 7			Model 8		
	OR	95% CI	*p*	OR	95% CI	*p*

TMAO	1.36	1.01–1.84	0.04	1.37	1.01–1.86	0.05
Choline				1.07	0.89–1.28	0.48
L-Carnitine				1.01	0.99–1.03	0.23
Betaine				0.98	0.93–1.03	0.36

Male						
	Model 9			Model 10		
	OR	95% CI	*p*	OR	95% CI	*p*

TMAO	1.38	0.93–2.04	0.11	1.33	0.92–1.93	0.13
Choline				1.08	0.79–1.48	0.64
L-Carnitine				1.02	0.99–1.05	0.20
Betaine				1.00	0.93–1.08	0.93

Female						
	Model 11			Model 12		
	OR	95% CI	*p*	OR	95% CI	*p*

TMAO	1.25	0.74–2.12	0.40	1.14	0.65–2.01	0.65
Choline				1.10	0.86–1.41	0.46
L-Carnitine				0.99	0.93–1.05	0.78
Betaine				0.94	0.86–1.02	0.15

OR, odds ratio. 95% CI, 95% confidence interval. OR shown were for plasma TMAO, choline, L-carnitine, and betaine in all, male, and female participants. Models 7, 9, and 11 adjusted for age, gender, BMI, Glu, TG, and Cr. Models 8, 10, and 12 adjusted for all factors in models 7, 9, and 11 plus choline, L-carnitine, and betaine.

**Table 4:** AUC of TMAO, choline, L-carnitine, and betaine for predicting CAD in all, male, and female participants.

	AUC	95% CI	*p*
All			
TMAO	0.6	0.52–0.69	0.03
TMAO + choline + L-carnitine + betaine	0.64	0.56–0.72	<0.01
Male			
TMAO	0.64	0.51–0.77	0.04
TMAO + choline + L-carnitine + betaine	0.64	0.52–0.77	0.04
Female			
TMAO	0.57	0.46–0.69	0.21
TMAO + choline + L-carnitine + betaine	0.64	0.53–0.75	0.02

**Table 5:** AUC of TMAO, choline, L-carnitine, and betaine for predicting severe artery stenosis in all, male, and female participants.

	AUC	95% CI	*p*
All			
TMAO	0.62	0.52–0.72	0.02
TMAO + choline + L-carnitine + betaine	0.66	0.56–0.75	<0.01
Male			
TMAO	0.64	0.51–0.78	0.05
TMAO + choline + L-carnitine + betaine	0.64	0.5–0.77	0.05
Female			
TMAO	0.58	0.42–0.74	0.26
TMAO + choline + L-carnitine + betaine	0.68	0.53–0.83	0.01

## Data Availability

The data used to support the findings of this study are included within the tables of the paper.

## Supplementary Materials

Supplementary Materials Supplemental Table 1: distribution of TMAO, choline, L-carnitine, and betaine in different gender with or without CAD.
