# Supplementary material for: The Association between Trimethylamine N-Oxide and Its Predecessors Choline, L-Carnitine, and Betaine with Coronary Artery Disease and Artery Stenosis
Source: Cardiol Res Pract. 2020 Aug 13;2020:5854919. doi: 10.1155/2020/5854919 (PMC7443013; doi:10.1155/2020/5854919)
Supplement: Supplementary Materials — Supplemental Table 1: distribution of TMAO, choline, L-carnitine, and betaine in different gender with or without CAD. [file 5854919.f1.docx]

Supplemental table

**Supplemental table 1. Distribution of TMAO, choline, l-carnitine and betaine in different gender with or without CAD.**

|  | CON | CAD | *p* |
| --- | --- | --- | --- |
| Male | N=31 | N=40 |  |
| TMAO (μM) | 1.22(0.8-1.62) | 1.65(1.04-2.84) | 0.04 |
| Choline (μM) | 8.67(6.97-10.76) | 8.71(7.36-9.67) | 0.85 |
| L-carnitine (μM) | 43.89(36.12-51.68) | 42.74(35.87-48.67) | 0.61 |
| Betaine (μM) | 37.43(29.43-40.79) | 33.48(25.6-42.49) | 0.43 |
| Female | N=42 | N=54 |  |
| TMAO (μM) | 1.11(0.61-1.82) | 1.33(0.77-2.09) | 0.21 |
| Choline (μM) | 8.12(6.71-9.32) | 8.17(6.52-9.5) | 0.98 |
| L-carnitine (μM) | 43.48(33.08-49.41) | 44.65(34.52-49.08) | 0.68 |
| Betaine (μM) | 37.68(29.37-42.5) | 32.41(26.79-41.21) | 0.09 |

Continuous data are presented as median (interquartile range).
